# NanoFN10: A High-Contrast Turn-On Fluorescence Nanoprobe for Multiphoton Singlet Oxygen Imaging

**DOI:** 10.3390/s23104603

**Published:** 2023-05-09

**Authors:** Renzo P. Zanocco, Roger Bresolí-Obach, Francisco Nájera, Ezequiel Pérez-Inestrosa, Antonio L. Zanocco, Else Lemp, Santi Nonell

**Affiliations:** 1 Departamento de Química Orgánica y Fisicoquímica, Facultad de Ciencias Químicas y Farmacéuticas, Universidad de Chile, Santiago 8330015, Chile; 2 Institut Químic de Sarrià, Universitat Ramon Llull, Via Augusta 390, 08017 Barcelona, Spain; 3 Departamento de Química Orgánica, Universidad de Málaga, Campus Teatinos s/n, 29071 Málaga, Spain; 4 Instituto de Investigación Biomédica de Málaga y Plataforma en Nanomedicina—IBIMA, Plataforma Bionand, Parque Tecnológico de Andalucía, 29590 Málaga, Spain

**Keywords:** singlet oxygen, fluorescent nanoprobe, multiphoton excitation

## Abstract

An “off-on” fluorescent nanoprobe for near-infrared multiphoton imaging of singlet oxygen has been developed. The nanoprobe comprises a naphthoxazole fluorescent unit and a singlet-oxygen-sensitive furan derivative attached to the surface of mesoporous silica nanoparticles. In solution, the fluorescence of the nanoprobe increases upon reaction with singlet oxygen both under one- and multiphoton excitation, with fluorescence enhancements up to 180-fold. The nanoprobe can be readily internalized by macrophage cells and is capable of imaging intracellular singlet oxygen under multiphoton excitation.

## 1. Introduction

Singlet molecular oxygen (O_2_(^1^Δ_g_), hereafter ^1^O_2_), the first electronic excited state of the oxygen molecule [1,2], is a highly reactive oxygen species (ROS) capable of oxidizing electron-rich cellular components such as proteins, nucleic acids, and lipid structures [3]. ^1^O_2_ plays a key role in a wide variety of chemical and biological processes, e.g., in organic synthesis [4], photodynamic therapy of cancer [5], the eradication of pathogenic microorganisms [6], cell signaling [7], or plant defense [8]. 

Notwithstanding its relevance, only a few techniques are available for monitoring ^1^O_2_ in biological media [2]. The preferred one is the optical detection of its near-infrared phosphorescence (NIR) at 1270 nm, which is selective, non-invasive, and robust [9,10]. Nonetheless, the detection of NIR phosphorescence requires sophisticated equipment available only in a few specialized laboratories [11]. In addition, the ^1^O_2_ phosphorescence quantum yield is exceedingly low in water (<1 × 10^−^^6^, meaning that only one in a million ^1^O_2_ molecules decays by emitting a photon) and even lower in biological media due to the action of ^1^O_2_ quenchers. Hence, the detection of this reduced number of photons poses severe technical challenges. Not surprisingly, alternative, simpler, yet indirect detection methods have been developed based on probes that react selectively with ^1^O_2_ [12,13,14].

Among them, fluorescent probes are attractive tools for the detection of ^1^O_2_ in biological media due to their high sensitivity and fast response, together with the widespread adoption of fluorescence microscopy and imaging techniques [15,16,17,18]. The most popular fluorescence probes use anthracenes to trap ^1^O_2_, e.g., anthracene dipropionic or dimalonic acids (ADPA and ADMA, respectively) [19] or Singlet Oxygen Sensor Green (SOSG) [20]. Anthracenes react with ^1^O_2_, forming endoperoxides and losing their native fluorescence as a result. ADPA and ADMA are thus turn-off probes. In contrast, SOSG, composed of an anthracene bound to a fluorescein-like fluorophore, is a turn-on probe whose fluorescence (due to fluorescein) is quenched by the intact anthracene moiety in its native state. Oxidation of anthracene by ^1^O_2_ leads to fluorescence enhancements up to 10-fold [15]. A negative effect of the presence of anthracenes in both probes is their non-negligible ability to self-photosensitize the production of ^1^O_2_, which may lead to false positives if appropriate control experiments are not employed [21]. In addition, molecular probes readily form complexes with proteins, which inhibit their internalization by cells and lead to strong fluorescence enhancements. This decreases the contrast between the native and oxidized forms, hampering the ability to detect ^1^O_2_ [22,23]. We have recently shown that conjugation of ADPA and SOSG to nanoparticles minimizes binding to proteins, thereby allowing the detection of ^1^O_2_ in bacteria [24] and in eukaryotic cells [25]. Moreover, we have recently described a new class of ^1^O_2_ fluorescent molecular probes, the furyl naphthoxazoles (FNs), which achieve fluorescence enhancements up to 300-fold owing to a change in absorption and fluorescence spectrum upon reaction with ^1^O_2_ [26]. In this work, we have combined the two approaches to develop a furyl-naphthoxazole nanoprobe (termed NanoFN10) that brings together the high contrast of FN probes and the advantages of the nano approach to improve the detection of ^1^O_2_ in biological media. 

## 2. Materials and Methods

### 2.1. General Materials

The following were purchased from Sigma-Aldrich (St. Louis, MO, USA) and used as received: 2-Methylnaphthoxazole, 5-(hydroxymethyl)furan-2-carbaldehyde, new methylene blue (NMB), rose bengal (RB), tetraethyl orthosilicate (TEOS), cetyltrimethylammonium chloride solution (25 wt % in H_2_O; CTAC), 3-(triethoxysilyl)propyl isocyanate, (3-iodopropyl)trimethoxysilane, 4,7,10-trioxa-1,13-tridecanediamine (PEG-diamino linker), succinic anhydride, *N*-(3-dimethylaminopropyl)-*N*′-ethylcarbodiimide hydrochloride (EDC), *N*-hydroxysuccinimide (NHS), 4-(dimethylamino)pyridine (DMAP), Dubelcco’s phosphate-buffered saline (PBS), quinine sulfate (QS), 9,10-dimethyl anthracene (DMA), dimethyl sulfoxide (DMSO), phenalenone (PN), potassium superoxide (potassium dioxide, KO_2_), and hydrogen peroxide (H_2_O_2_). Absolute ethanol (EtOH), benzene, acetonitrile (ACN), dimethylformamide (DMF), ammonia solution (NH_3_ 30 wt % in H_2_O), dichloromethane (DCM), hydrochloric acid solution (HCl 37 wt % in H_2_O), glacial acetic acid (AcOH), potassium hydroxide (KOH), and methanol (MeOH) were supplied by Panreac (Barcelona, Spain). Roswell Park Memorial Institute (RPMI) 1640 medium, fetal bovine serum, penicillin, streptomycin, and gentamicin were purchased from Life Technologies, Invitrogen, (Carlsbad, CA, USA).

### 2.2. General Synthetic Remarks

All reactions were monitored by TLC analysis using POLYGRAM^®^ SIL G/UV254 pre-coated polyester sheets (0.2-mm thickness) (Machery-Nagel, Düren, Germany).

NMR spectra were recorded at room temperature on a Varian Mercury 400 (^1^H at 400 MHz and ^13^C at 100.6 MHz; Palo Alto, CA, USA) spectrometer. The chemical shifts (δ) are reported in parts per million (ppm) relative to the solvent signal, and coupling constants (J) are reported in Hertz (Hz). The following abbreviations are used to define the multiplicities in ^1^H NMR spectra: s = singlet, d = doublet, t = triplet, q = quartet, dt = doublet of triplets, ddd = doublet of doublet of doublets, m = multiplet, or combinations of these descriptive names.

Infrared spectra were recorded in a Nicolet Magna 560 FTIR (Madison, WI, USA) spectrophotometer supported on a potassium bromide disk. Values are reported in wave numbers (cm^−1^).

Mass spectrometry (MS) for reported compounds was conducted on an Agilent Technologies 5975 mass spectrometer (Santa Clara, CA, USA) operating in electron ionization (EI) mode at 70-eV and 4-kV accelerating potential.

### 2.3. Synthesis of FN10 [(E)-(5-(2-(Naphtho[1,2-d]oxazol-2-yl)vinyl)furan-2-yl)methanol] 

To a mixture of 2-methylnaphthoxazole (1.1 mmol, 170 μL) and 5-(hydroxymethyl)furan-2-carbaldehyde (1.0 mmol, 100 μL) in 2 mL of dimethyl sulfoxide, 62 μL of aqueous KOH 50% was added. The mixture was stirred for 1 h at room temperature protected from light. The addition of 10 mL of water afforded a yellow precipitate, which was washed with 50 mL of cold water and 80 mL of cold methanol. Recrystallization from acetonitrile afforded 131 mg of the yellow product (FN10), yield 45%. ^1^H-NMR (400 MHz, DMSO-d_6_): 8.36 (d, 1H, J = 8.5 Hz, Ar-H_8_), 8.09 (d, 1H, J = 8.0 Hz, Ar-H_5_), 7.93 (d, 1H, J = 9.0 Hz, Ar-H_3_), 7.89 (d, 1H, J = 9.0 Hz, Ar-H_4_), 7.69 (dt, 1H, J = 8.0; 7.0 Hz, Ar-H_7_), 7.62 (d, 1H, J = 16.0 Hz, Vi-H_a_), 7.58 (dt, 1H, J = 8.0; 7.0 Hz, Ar-H_6_), 6.92 (d, 1H, J = 15.0 Hz, Vi-H_b_), 6.89 (d, 1H, J = 3.5 Hz, Fu-H_c_), 6.47 (d, 1H, J = 3.5 Hz, Fu-H_d_), 4.47 (s, 2H, -CH2-), 3.52 (s, 1H, -OH). ^13^C-NMR (100 MHz, DMSO-d_6_): 162.2, 158.6, 150.7, 147.7, 137.5, 131.3, 129.3, 127.7, 126.7, 126.0, 126.0, 125.9, 122.0, 115.9, 111.4, 110.4, 110.3, 56.3. IR (KBr) υ (cm^−1^): 3293, 3062, 2922, 1633, 1372, 1018, 950. MS: calculated: 292.09 Da; experimental: 292.12 Da.

### 2.4. Isolation and Identification of the FN10 Oxidation Product (FN10-ox)

Fifty mg of FN10 was dissolved in 30 mL of methanol. Afterward, 50 mL of a methanolic new methylene blue solution (3.1 mM) was added and irradiated with red light (660 ± 10 nm; M660L4, Thorlabs, Newton, NJ, USA) for 24 h. The photoproduct was purified by silica flash chromatography (ethyl acetate/hexane 1:10 as mobile phase). ^1^H-NMR (300 MHz, DMSO-d_6_): 8.37 (d, 1H, J = 8.0 Hz, Ar-H_9_), 8.11 (d, 1H, J = 8.0 Hz, Ar-H_12_), 7.97 (d, 1H, J = 9.0 Hz, Ar-H_8_), 7.90 (d, 1H, J = 9.0 Hz, Ar-H_7_), 7.81 (d, 1H, J = 5.5 Hz, Vi-H_3_), 7.71 (ddd, 1H, J = 8.0; 6.0; 1.0 Hz, Ar-H10), 7.61 (ddd, 1H, J = 8.0; 5.5; 1.0 Hz, Ar-H_11_), 6.98 (d, 1H, J = 17.5 Hz, Vi-H_5_), 6.60 (d, 1H, J = 5.5 Hz, Vi-H_4_), 6.26 (d, 1H, J = 17.5 Hz, Ar-H_5_), 3.76 (m, 2H, OC-H2), 3.35 (s, 3H, OC-H1); ^13^C-NMR (100 MHz, CDCl_3_): 189.0, 152.7, 128.7, 128.6, 128.0, 127.6, 127.4, 127.3, 125.9, 125.7, 123.8, 122.2, 122.0, 120.5, 110.6, 107.3, 103.7, 60.7, 15.2. IR (KBr) υ (cm^−1^); 3334, 3067, 2925, 1773, 1622, 1445, 1083. MS: calculated: 322.10 Da; experimental: 322.20 Da. 

### 2.5. Synthesis of the Silylated PEG-Amino Linker [(EtO)_3_Si-L-NH_2_)]

*N*-(4,7,10-trioxa-13-tridecaneamine)-N’(3-triethoxysilyl)propyl-urea was synthesized as described previously [25].

### 2.6. Synthesis of Silylated Rose Bengal [(MeO)_3_Si-RB: 2,4,5,7-Tetraiodo-3-oxo-9-(2,3,4,5-tetrachloro-6-((3-(trimethoxysilyl)-propoxy)-carbonyl)-phenyl)-3H-xanthen-6-olate]

Rose bengal (51 mg, 53 μmol) was added to a solution of (3-iodopropyl)trimethoxysilane (30 μL, 153 μmol) in 1 mL of DMF anhydrous. The solution was stirred for 2 h at 90 °C. Afterward, the crude was used without further purification.

### 2.7. Synthesis of Mesoporous Silica Nanoparticles (MSNP)

MSNPs were synthesized as described previously [25,27] and stored suspended in absolute EtOH (10 mg/mL).

### 2.8. Grafting of Rose Bengal onto Mesoporous Silica Nanoparticles (MSNP-RB)

One mL of the (MeO)_3_Si-RB solution was added to 12 mL of MSNP solution (33 mg/mL). The solution was left stirring at room temperature for 24 h. Afterward, the MSNP-RB was recovered by centrifugation (15,000× *g*; 20 min). The MSNP was washed with EtOH. This procedure was repeated several times until no color was observed in the supernatant. The final MSNP-RB nanoparticles were stored suspended in absolute EtOH (10 mg/mL).

### 2.9. Grafting of the Sylilated PEG-Amino Linker onto Mesoporous Silica Nanoparticles

Two hundred mg of (EtO)_3_Si-L-NH_2_ (430 μmols) were added to 12 mL of MSNP or MSNP-RB suspension (33 mg/mL) and left stirring at room temperature for 24 h. Afterward, the nanoparticles were recovered by centrifugation (15,000× *g*; 20 min). The nanoparticles were washed twice with ethanol and once with ACN. The final MSNP-L-NH_2_ or RB-MSNP-L-NH_2_ nanoparticles were stored suspended in ACN (10 mg/mL).

### 2.10. Grafting of Carboxylic-Acid Groups on the MSNP-L-NH-CO-CH_2_-CH_2_-COOH and RB-MSNP-L-NH-CO-CH_2_-CH_2_-COOH Nanoparticles

One hundred mg of succinic anhydride (1 mmol) was added to 12 mL of MSNP-L-NH_2_ or RB-MSNP-L-NH_2_ suspension (33 mg/mL). The suspensions were left stirring at room temperature for 24 h. Afterward, the nanoparticles were recovered by centrifugation (15,000× *g*; 20 min) and washed thrice with ACN. The final MSNP-L-NH-CO-CH_2_-CH_2_-COOH or RB-MSNP-L-NH-CO-CH_2_-CH_2_-COOH nanoparticles were stored suspended in ACN (10 mg/mL).

### 2.11. Synthesis of NanoFN10 [MSNP-L-NH-CO-CH_2_-CH_2_-CO-O-FN10] and NanoFN10RB [MSNP-L-NH-CO-CH_2_-CH_2_-CO-O-FN10]

Three hundred and fifty mg (2.3 mmol) of EDC, 350 mg of NHS (3.3 mmol), and 300 mg (2.5 mmol) of DMAP were directly dissolved into 12 mL of a suspension of MSNP-L-NH-CO-CH_2_-CH_2_-COOH or RB-MSNP-L-NH-CO-CH_2_-CH_2_-COOH nanoparticles (ACN, 33 mg/mL). The suspension was left stirring at room temperature for 2 h. Then, 100 mg of FN10 was added and left stirring for an additional 72 h. Afterward, the nanoparticles were recovered by centrifugation (15,000× *g*; 20 min) and washed twice with ACN and twice with MeOH. The final nanoFN10 and nanoFN10RB nanoparticles were stored suspended in MeOH (5 mg/mL).

### 2.12. Determination of the Hydrodynamic Size, ζ-Potential, and Organic Elemental Analysis of the Synthesized Nanoparticles

Hydrodynamic size and *ζ*-potential of the synthesized (MSNPs) were measured using a Nano-ZS Zetasizer (Malvern Instruments Ltd., Worcestershire, UK). For hydrodynamic size, a diluted aliquot in EtOH was measured. For *ζ*-potential examination, a diluted aliquot in milli-Q water was measured. 

### 2.13. One-Photon Spectroscopy

Absorption spectra were recorded on a Cary 6000i spectrophotometer (Varian, Palo Alto, CA, USA) and a Perkin Elmer Lambda 2B spectrophotometer (Norwalk, CT, USA). Fluorescence emission spectra were recorded using a Spex Fluoromax-4 spectrofluorometer (Horiba Jobin-Ybon, Edison, NJ, USA) and an Optical Building Blocks Quatro III spectrofluorometer (Birmingham, NY, USA). 

Time-resolved near-infrared phosphorescence experiments were carried out using a customized Fluotime 200 fluorescence lifetime system (PicoQuant GmbH, Berlin, Germany). Briefly, a diode-pumped Nd:YAG laser (FTSS355-Q, Crystal Laser, Berlin, Germany) was used for excitation at 355 or 532 nm (10 kHz, 0.5 or 1.2 μJ per pulse, respectively). A 1064-nm rugate notch filter (Edmund Optics Ltd., Barrington, NJ, USA) was placed at the exit port of the laser to remove any residual component of its fundamental emission in the NIR region. The luminescence exiting from the side of the sample was filtered by one long-pass filter of 1000 nm to remove any scattered laser radiation and isolate the NIR emission. Additional narrow bandpass filters at 1270, 1220, or 1110 nm were used to select a particular near-infrared region. A TE-cooled Hamamatsu NIR sensitive photomultiplier tube assembly (H9170-45, Hamamatsu Photonics, Hamamatsu City, Japan) was used as the detector. Photon counting was achieved with a multichannel scaler (PicoQuant’s NanoHarp 250; PicoQuant GmbH, Berlin, Germany).

### 2.14. Two-Photon Spectroscopy

FN10 was analyzed using a Leica SP5 MP scanning confocal/multiphoton system with a Leica (Wetzler, Germany) DM6000 inverted microscope and 10× HCX PL APO CS 10× 0.40 NA dry objective lens with the Spectra Physics Ti:Saphire MaiTai HP tunable IR pulsed laser (MKS Instruments, Andover, MA, USA) as the light source. Samples were loaded into thin quartz cuvettes and positioned to focus on the air–solvent interface. Focusing at this interface provides a negative control for non-specific reflections and background noise. FN10 was characterized by using excitation and emission scans acquired using the Leica LAS AF software suite. 

Excitation scans were performed using a fixed emission detection window (330–550 nm) and variable multiphoton excitation (700–1040 nm in 10 nm intervals). Average intensity was measured in regions of interest (ROIs) positioned in sample and air regions of each assay image. FN10 was photooxidized by adding MSNP-RB and irradiating with green light. After the irradiation, MSNP-RB was removed by centrifugation (15,000× *g*; 10 min).

### 2.15. Cell Culture

N13 microglia adherent cells were grown to 60% confluence in a Labtek 8 well-chambered slide in standard culture medium (RPMI media with 10% Fetal Bovine Serum, 1% Pen/Strep 10,000 units/mL, 1% Gentamicin) and under standard conditions (37 °C and 5% CO_2_ in a humidified incubator) and incubated nanoFN10RB for 24 h. Cells were washed three times with fresh culture medium to remove nanoFN10RB in the remaining medium before transfer to the microscope.

### 2.16. Microscopy Analysis

Multiphoton experiments employed a Leica SP5 MP scanning confocal/multiphoton system using a Leica DM6000 inverted microscope and HCX IRAPO L x25.0 NA0.95 WATER objective lens with Spectra Physics MaiTai HP tunable IR-pulsed and 561-nm CW lasers as light sources. N13 microglia cells within the chambered slide were maintained under optimal conditions (37 °C and 5% CO_2_ in a semi-closed, humidified microscope incubator) throughout the experiment. 

Experiments were performed using a sequential time-lapse mode at a single focal plane. Samples were alternately measured for fluorescence using the multiphoton laser tuned to 720 nm (1% power with 3 accumulations) and a 413–532-nm detection window, and then activated by scanning the sample a total of 50 times using the 561-nm laser with the transmission set to between 0% and 8% maximum power. Each complete cycle of detection and activation was completed in 27 s and immediately followed by the next cycle. Conditions were chosen to allow photooxidized molecules to be detected while minimizing the photobleaching effect of the multiphoton laser and maximizing the ^1^O_2_ generation. All photographs were processed and analyzed using the Fiji J software (Adobe Systems, San Jose, CA, USA) [28,29].

## 3. Results and Discussion

### 3.1. Synthesis and Characterization of the Molecular Probe FN10

**Figure 1:**
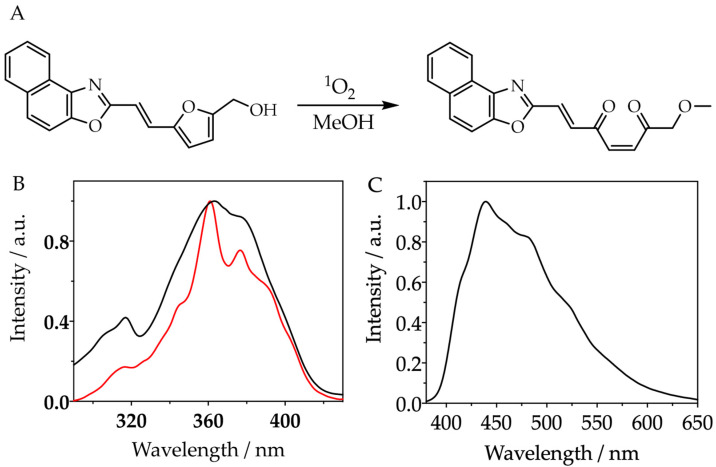
(**A**): Synthesis of FN10. (**B**): Normalized absorption (black line) and fluorescence excitation (red line) spectra of FN10 in methanol. (**C**): Normalized fluorescence spectrum.

The molecular probe FN10 is a modification of the furyl naphthoxazoles reported in our previous communication [26], in which the hydrogen in position C-5 of the furan has been replaced by a hydroxymethyl group. This improves the furan’s reactivity towards ^1^O_2_ and allows its conjugation to nanoparticles via a Steglich esterification [30,31]. FN10 has been synthesized by a one-pot, two-step reaction, in which the conjugation of methylnaphthoxazole anion to furaldehyde is followed by a dehydration step (Figure 1A). The spectroscopic characterization is shown in the supplementary information (Figure S1).

The absorption spectrum of FN10 in methanol shows a maximum at 363 nm and a second peak at 290 nm, while the fluorescence spectrum shows a maximum at 439 nm (Figure 1B). The fluorescence quantum yield (Φ_F_) ranges from 0.05 in acetonitrile to 0.005 in methanol (Figure S2), indicating efficient quenching of the naphthoxazole singlet excited state by the furan in the protic solvent. The photophysical properties of FN10 in methanol are collected in Table 1.

### 3.2. Reactivity of FN10 with Singlet Oxygen

**Figure 2:**
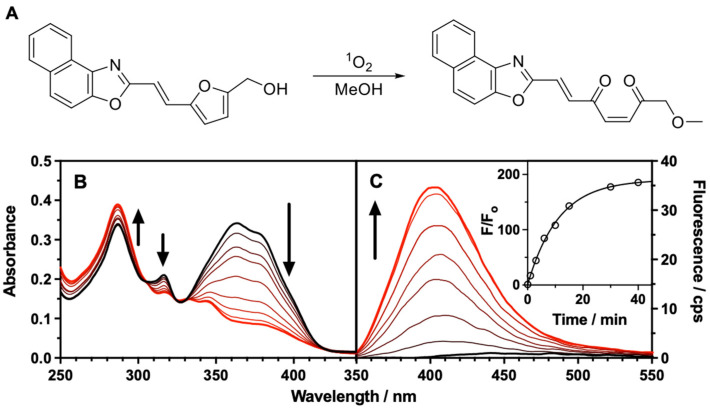
(**A**): Oxidation of FN10 with ^1^O_2_. (**B**): Absorbance changes upon the reaction of FN10 with ^1^O_2_. (**C**): Fluorescence changes upon the reaction of FN10 with ^1^O_2_. Inset: Fluorescence intensity enhancement at 400 nm. Note the blue shift and strong fluorescence increase. ^1^O_2_ was generated by photosensitization with new methylene blue (5 μM at 640 ± 10 nm). The experiments were carried out in aerated methanol. The fluorescence was excited at 335 nm to produce the highest enhancement.

**Figure 3:**
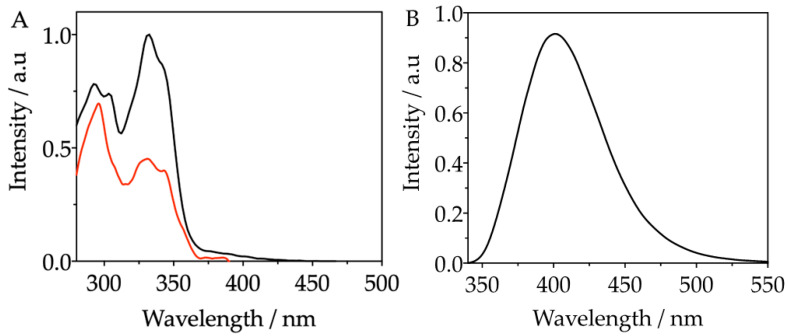
Spectra of FN10-ox in methanol. (**A**): Normalized absorption (black line) and excitation spectra (red line). (**B**): Fluorescence spectrum.

The reactivity of FN10 towards ^1^O_2_ was studied by generating it using new methylene blue photosensitization and monitoring the concomitant changes in the absorption and emission spectra of the probe (Figure 2). In the presence of ^1^O_2_, the absorbance of the 363-nm FN10 band decreased strongly (Figure 2B), and an intense fluorescence emission appeared with a maximum at 400 nm (Figure 2C). HPLC analysis of the photoirradiated samples revealed the formation of a single photoproduct, FN10-ox (Figure S3). The observation of isosbestic points in the absorption spectra is consistent with this finding. The structure of FN10-ox was determined by MS, IR, and ^1^H/^13^C-NMR spectroscopies (Figure S4) and is shown in Figure 2A. Furan oxidation eliminates the fluorescence quencher and shortens the conjugation path in the probe, leading to changes in the absorption and emission spectra that enable the selective excitation and detection of FN10-ox fluorescence (Figure 3). The excitation spectra of the photooxidized samples show a maximum at 335 nm (Figure S5). Exciting the samples at this wavelength leads to a remarkable fluorescence enhancement of 180-fold when observed at 400 nm (inset of Figure 2C).

The rate constant for overall (physical and reactive) ^1^O_2_ quenching by FN10 was determined by measuring the ^1^O_2_ decay rate as a function of FN10 concentration in time-resolved ^1^O_2_ phosphorescence assays (Figure S6A) [32]. The value found, 3.0 × 10^7^ M^−^^1^ s^−^^1^ (Figure S6B), is 32-fold larger than for unsubstituted furyl naphthoxazoles [26]. The reactive rate constant was determined by comparing the rate of photooxidation of FN10 with that of dimethyl anthracene [32] and is 1.7 × 10^7^ M^−^^1^ s^−^^1^ (Figure S6C), comparable to that of the most reactive furans [33]. The reactivity of FN10 towards other ROS was examined, and it was found that it does not react with superoxide or hydrogen peroxide (Figure S7). Finally, FN10 shows a negligible quantum yield of ^1^O_2_ self-sensitization (Φ_Δ_ = 0.003 in methanol and acetonitrile, Figure S8); for comparison, SOSG has Φ_Δ_ = 0.03 at 355 nm and 0.009 at 532 nm in methanol [21]. Taken together, the above results suggest that FN10 could be an excellent fluorescent probe for ^1^O_2_ detection.

### 3.3. Synthesis and Characterization of the Nanoprobe NanoFN10

**Scheme 1:**
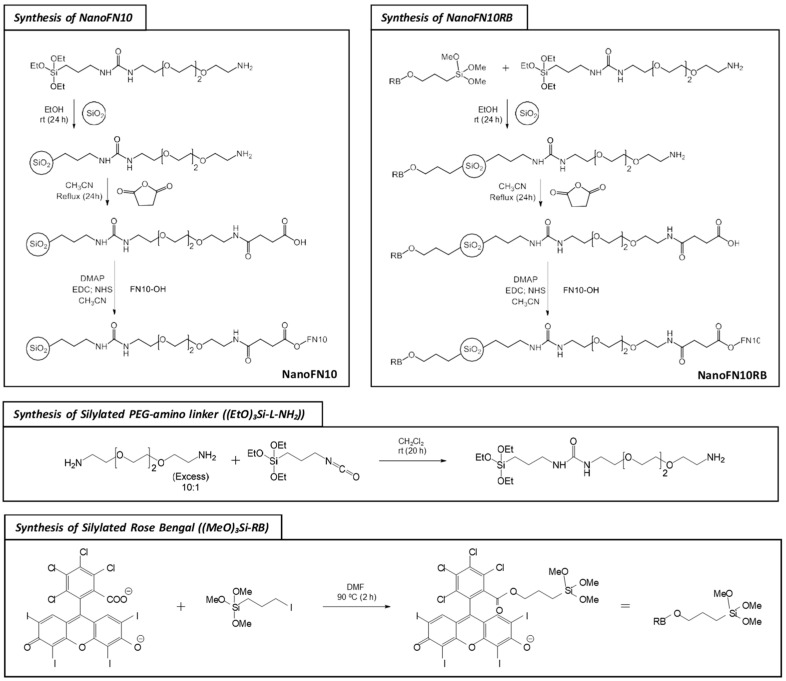
Synthesis and derivatization of NanoFN10 and NanoFN10RB.

Given the excellent properties of FN10, we aimed to synthesize a nanosensor (nanoFN10) where FN10 would be covalently linked to the surface of mesoporous silica nanoparticles through a 2.3-nm PEG linker (Figure S9). Previous results from our laboratory show that linkers of this length preclude the complexation of the probe with proteins while still preserving its reactivity towards ^1^O_2_ (Scheme 1) [24,25]. 

In the first step, 10 eq of the diamino PEG linker was reacted with 1 eq of (3-isocyanate propyl)-triethoxysilane to yield a monosubstituted aminosilylated-PEG linker, which was then grafted onto the surface of 160-nm mesoporous silica nanoparticles. Since the amino moieties cannot react directly with the hydroxyl group of FN10, they were converted to carboxylic moieties via reaction with succinic anhydride. Finally, the FN10 fluorescent probe was conjugated to the functionalized nanoparticles by Steglich esterification. The bare MSNPs and the final nanoFN10 probe were characterized by their hydrodynamic diameter (150 to 200 nm) and *ζ*-potential (−21 to −23 mV). Covalent conjugation of FN10 did not significantly modify the size and the *ζ*-potential of the silica nanoparticles.

**Figure 4:**
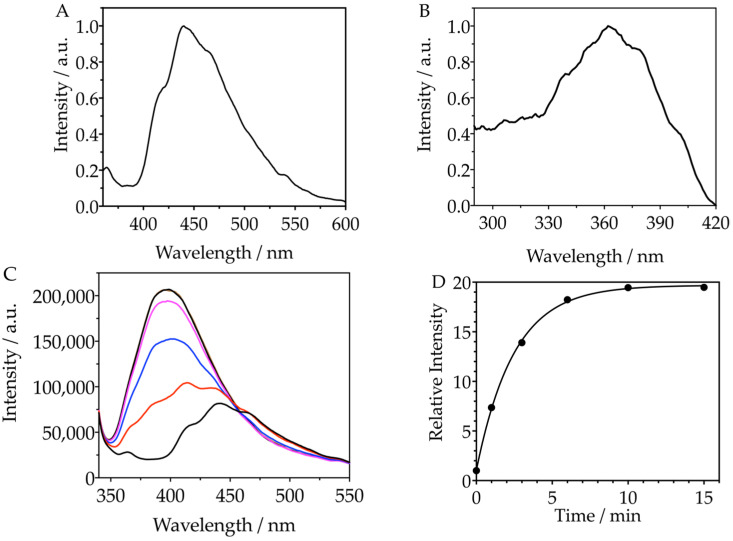
(**A**): Normalized emission spectra of nanoFN10 (λ_exc_ = 335 nm). (**B**): Normalized excitation spectra of nanoFN10 (λ_obs_ = 450 nm). (**C**): NanoFN10 emission spectra changes upon reaction with ^1^O_2_ (λ_exc_ = 335 nm). The photosensitizer used was new methylene blue 5 μM, the irradiation wavelength was 640 ± 10 nm, and the experiment was carried out in aerated methanol. (**D**): NanoFN10 fluorescence enhancement kinetics at λ_obs_ = 400 nm.

Once the nanoFN10 was in hand, the particles were suspended in aerated methanol for optical and photophysical characterization. The emission and excitation spectra of nanoFN10 were essentially identical to those of its molecular counterpart, although 10-nm blue-shifted probably due to the esterification of the hydroxymethyl group (Figure 4A,B). Irradiation of the suspension with red light in the presence of added new methylene blue led to the expected growth of a blue-shifted, bright fluorescence band, as in the case of FN10. A fluorescence enhancement of 20-fold was observed at 390 nm (Figure 4C,D).

Having established that the nanoprobe enhances its fluorescence upon reaction with ^1^O_2_, we prepared a new nanosystem by simultaneously grafting the sylilated diamino PEG linker and a sylilated ^1^O_2_ photosensitizer (rose bengal, RB) to its surface, thereby enabling orthogonal attachment of FN10 and RB (Scheme 1). This ensured the generation of ^1^O_2_ in close vicinity to the sensor in the cellular experiments. The bifunctional nanoprobe (termed nanoFN10RB) reproduced the behavior of nanoFN10 (Figure S10).

### 3.4. Imaging of Singlet Oxygen in Cells

We have successfully developed a novel turn-on singlet oxygen-specific fluorescent probe that shows a remarkable contrast between the native and oxidized forms. We have attached it to the surface of mesoporous silica nanoparticles and combined it with a photosensitizer to ensure co-localization with the singlet oxygen source. However, an excitation wavelength of 335 nm is required to achieve the maximum fluorescence enhancement upon reaction with ^1^O_2_, which is not suitable for studies in biological media. Notwithstanding, the probe could be valuable for multiphoton imaging microscopy. Multiphoton excitation would confer additional advantages for bioimaging such as longer excitation wavelength, deeper tissue penetration, lower fluorescence background, reduced photodamage in living systems, and better three-dimensional spatial resolution [34,35]. In light of a recent report on a naphthalimide-anthracene fluorescent probe that showed multiphoton absorption properties [36], we investigated whether the fluorescence of FN10 could be amenable to multiphoton excitation.

**Figure 5:**
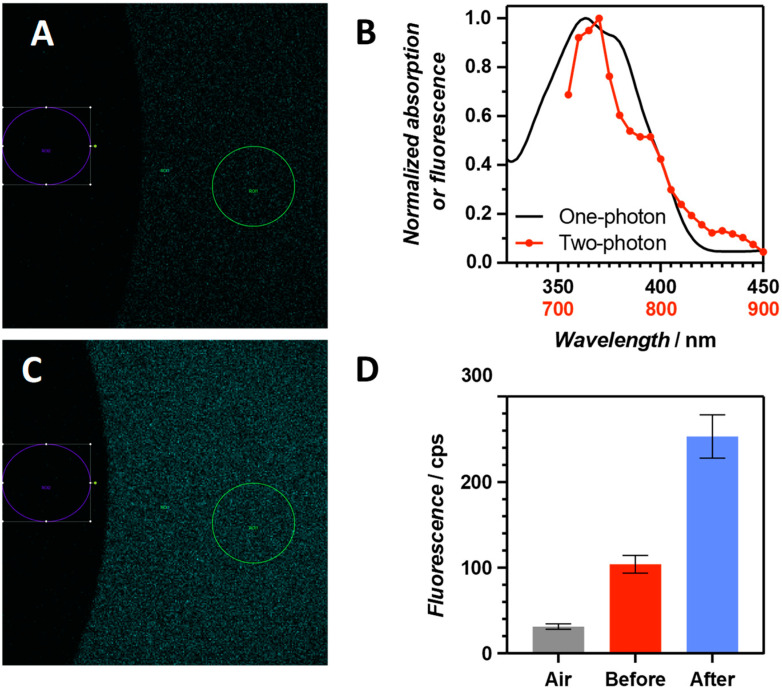
FN10 fluorescence images obtained by excitation with two-photon absorption (λ_exc_ = 720 nm; λ_obs_ = 450–540 nm) before (**A**) and after (**C**) reacting with ^1^O_2_. In both images, two regions are observed: the dark one on the left corresponds to an air bubble while the right one corresponds to FN10/FN10-ox methanolic solution. The circles indicate the region used to quantify the emission intensity. (**B**): Normalized FN10 one-photon absorption (black line) and two-photon fluorescence excitation (red line) spectra. (**D**): Two-photon fluorescence intensity corresponding to the air bubble, FN10 and FN10-ox (before and after reacting with ^1^O_2_, respectively).

As shown in Figure 5A, multiphoton excitation was able to elicit the fluorescence of FN10 in microscopy imaging experiments using a simple solution with an air bubble as a zero-contrast reference image. Figure 5B shows the one-photon absorption and the two-photon fluorescence excitation spectra of FN10, the latter derived from spectroscopic analysis of the microscopy images. The maximum of the two-photon excitation spectrum is found at exactly twice the maximum of the one-photon absorption (and excitation spectra), confirming that FN10 is amenable to two-photon excitation. 

Upon reaction with ^1^O_2_, a 3-fold increase in fluorescence was observed (Figure 5C,D). The contrast was much smaller than under one-photon excitation because the excitation wavelength for FN10-ox had to be set at 720 nm rather than 670 nm (=2 × 335 nm) due to equipment limitations. Despite the lower fluorescence enhancement, we attempted to image ^1^O_2_ in N13 microglia adherent cells. 

In the first series of experiments, the N13 microglia cells were incubated with the nanoFN10RB probe for 24 h, and images were taken under two-photon excitation at *λ*_exc_ 720 nm (Figure S11). As shown in Figure S12, the two-photon fluorescence colocalizes with the one-photon fluorescence of RB (*λ*_exc_ 561 nm), confirming its assignment to FN10.

**Figure 6:**
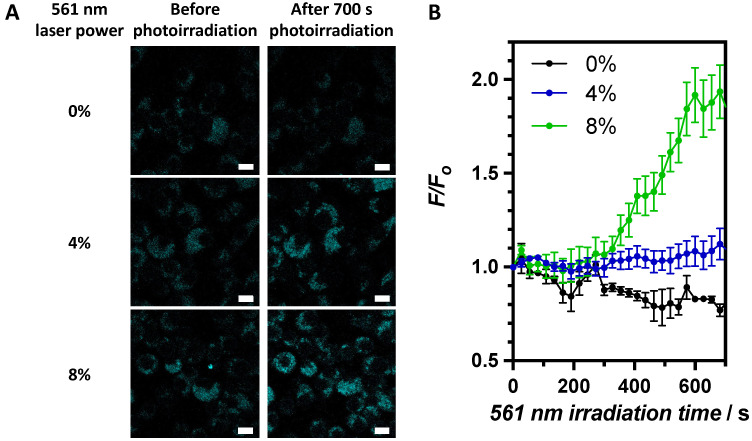
(**A**): Fluorescence images of N13 microglia cells incubated with nanoFN10RB. The images in the left column are before photoirradiation, and those in the right column were taken after 700-s photoirradiation using different 561-nm power laser intensities. The length of the scale bar is 10 μm. (**B**): NanoFN10RB fluorescence enhancement kinetics as a function of the 561-nm laser power intensity (0%, 4%, and 8%; black, blue, and green lines, respectively). Open bars represent the mean SD for 20 independent NP systems where only the mean pixel intensity of the images is considered.

In a second series of experiments, a group of cells preincubated with nanoFN10RB were irradiated with a CW green laser (*λ*_exc_ 561 nm) at different laser powers (0%, 4%, and 8%), and biphotonic fluorescence images (*λ*_exc_ 720 nm) were recorded every 30 s (Figure 6A). At 0% green laser power, when no ^1^O_2_ was produced, the only effect observed was the photobleaching of the nanoprobe upon prolonged two-photon excitation. However, when ^1^O_2_ was generated by green light irradiation of RB, the initial photobleaching was followed by a fluorescence enhancement phase (Figure 6). The duration and extension of the photobleaching and fluorescence-enhancement phases depend on the laser power. At the maximum power (8%), a fluorescence enhancement of 80% could be observed. The time delay between the photobleaching and the fluorescence enhancement is attributed to the period required to eliminate the endogenous ^1^O_2_ quenchers that compete with the probe oxidation, as has been previously reported for bacteria during a PDT treatment [24]. These results confirm the ability of nanoFN10RB to generate and detect ^1^O_2_ inside N13 cells by NIR two-photon microscopy.

## 4. Conclusions

We have developed a novel naphthoxazole-furan turn-on fluorescent probe, FN10, which is selective and highly reactive towards ^1^O_2_; it shows enhancement factors up to 180 and produces very little ^1^O_2_ by photosensitization, and its fluorescence can be excited by multiphoton processes. FN10 was successfully attached to MSNPs to protect it from interaction with proteins and other biological components. NanoFN10RB is readily internalized by N13 microglia cells and reacts with intracellularly generated ^1^O_2_, enabling two-photon fluorescence imaging of this reactive oxygen species. Work is currently in progress to further enhance the contrast between the native and oxidized nanoprobes.

## Figures and Tables

**Table 1:** Photophysical properties of FN10 and FN10-ox in methanol.

**FN10**	
λ_abs_/nm	363
ε/cm^−1^M^−1^	4.3 × 10^4^
λ_flu_/nm	439
Φ_F_	0.005
Φ_Δ_	0.003
*k_q_*/M^−1^s^−1^	3.0 × 10^7^
*k_r_*/M^−1^s^−1^	1.7 × 10^7^
**FN10-ox**	
λ_abs_/nm	335
λ_flu_/nm	405
Fluorescence enhancement	180 (at 400 nm)

## Data Availability

The data presented in this study are available in the article and Supplementary Materials.

## Supplementary Materials

The following supporting information can be downloaded at: https://www.mdpi.com/article/10.3390/s23104603/s1. Figure S1: Spectroscopic characterization of FN10. Figure S2: Determination of the FN10 fluorescence quantum yield. Figure S3: HPLC monitoring of FN10 oxidation by singlet oxygen. Figure S4: Spectroscopic characterization of FN10-ox. Figure S5: Evolution of the excitation spectra of FN10 upon reaction with singlet oxygen. Figure S6: Reactivity of FN10 with singlet oxygen. Figure S7: Reactivity of FN10 with other reactive oxygen species. Figure S8: Quantum yield of singlet oxygen production by FN10. Figure S9: Geometry of FN10 grafted onto the surface of a silica particle through a PEG linker. Figure S10: Fluorescence evolution of the nanoFN10RB probe upon reaction with singlet oxygen. Figure S11: Two-photon microscopy of N13 microglia cells incubated with nanoFN10RB. Figure S12: Co-localization of FN10 and rose bengal fluorescence in microglia cells incubated with nanoFN10RB. References [37,38,39,40,41,42] are cited in the Supplementary Information.

## References

[1] Nonell S., Flors C. (2016). Singlet Oxygen: Applications in Biosciences and Nanosciences.

[2] Ogilby P.R. (2010). Singlet oxygen: There is indeed something new under the sun. Chem. Soc. Rev..

[3] Davies M.J. (2003). Singlet oxygen-mediated damage to proteins and its consequences. Biochem. Biophys. Res. Commun..

[4] Kopetzki D., Lévesque F., Seeberger P.H. (2013). A continuous-flow process for the synthesis of artemisinin. Chem. Eur. J..

[5] Agostinis P., Berg K., Cengel K.A., Foster T.H., Girotti A.W., Gollnick S.O., Hahn S.M., Hamblin M.R., Juzeniene A., Kessel D. (2011). Photodynamic therapy of cancer: An update. J. Am. Cancer Soc..

[6] Wainwright M., Maisch T., Nonell S., Plaetzer K., Almeida A., Tegos G.P., Hamblin M.R. (2017). Photoantimicrobials—Are we afraid of the light?. Lancet Infect. Dis..

[7] Kim C., Meskauskiene R., Apel K., Laloi C. (2008). No single way to understand singlet oxygen signalling in plants. EMBO Rep..

[8] Flors C., Nonell S. (2006). Light and singlet oxygen in plant defense against pathogens:  Phototoxic phenalenone phytoalexins. Acc. Chem. Res..

[9] Salokhiddinov K.I., Byteva I.M., Dzhagarov B.M. (1979). Duration of luminescence of singlet oxygen in solutions with laser excitation. Opt. Spektrosk..

[10] Jiménez-Banzo A., Ragàs X., Kapusta P., Nonell S. (2008). Time-resolved methods in biophysics. 7. Photon counting vs. analog time-resolved singlet oxygen phosphorescence detection. Photochem. Photobiol. Sci..

[11] Nonell S., Braslavsky S.E., Packer L., Sies H. (2000). Chapter 4: Time-resolved singlet oxygen detection. Methods in Enzymology 310.

[12] Rabello B.R., Gerola A.P., Pellosi D.S., Tessaro A.L., Aparício J.L., Caetano W., Hioka N. (2012). Singlet oxygen dosimetry using uric acid as a chemical probe: Systematic evaluation. J. Photochem. Photobiol. A Chem..

[13] Umezawa N., Tanaka K., Urano Y., Kikuchi K., Higuchi T., Nagano T. (1999). Novel Fluorescent Probes for Singlet Oxygen. Angew. Chem. Int. Ed..

[14] Kálai T., Hideg E., Vass I., Hideg K. (1998). Double (fluorescent and spin) sensors for detection of reactive oxygen species in the thylakoid membrane. Free Radic. Biol. Med..

[15] Flors C., Fryer M.J., Waring J., Reeder B., Bechtold U., Mullineaux P.M., Nonell S., Wilson M.T., Baker N.R. (2006). Imaging the production of singlet oxygen in vivo using a new fluorescent sensor, Singlet Oxygen Sensor Green. J. Exp. Bot..

[16] Tanaka K., Miura T., Umezawa N., Urano Y., Kikuchi K., Higuchi T., Nagano T. (2001). Rational design of fluorescein-based fluorescence probes. Mechanism-based design of a maximum fluorescence probe for singlet oxygen. J. Am. Chem. Soc..

[17] Price M., Reiners J.J., Santiago A.M., Kessel D. (2009). Monitoring singlet oxygen and hydroxyl radical formation with fluorescent probes during photodynamic therapy. Photochem. Photobiol..

[18] Kim S., Tachikawa T., Fujitsuka M., Majima T. (2014). Far-red fluorescence probe for monitoring singlet oxygen during photodynamic therapy. J. Am. Chem. Soc..

[19] Kim S., Fujitsuka M., Majima T. (2013). Photochemistry of singlet oxygen sensor green. J. Phys. Chem. B.

[20] Lindig B.A., Rodgers M.A.J., Schaap A.P. (1980). Determination of the lifetime of singlet oxygen in water-d2 using 9,10-anthracenedipropionic acid, a water-soluble probe. J. Am. Chem. Soc..

[21] Ragàs X., Jiménez-Banzo A., Sánchez-García D., Batllori X., Nonell S. (2009). Singlet oxygen photosensitisation by the fluorescent probe Singlet Oxygen Sensor Green^®^. Chem. Commun..

[22] Gollmer A., Arnbjerg J., Blaikie F.H., Pedersen B.W., Breitenbach T., Daasbjerg K., Glasius M., Ogilby P.R. (2011). Singlet Oxygen Sensor Green®: Photochemical behavior in solution and in a mammalian cell. Photochem. Photobiol..

[23] Ruiz-González R., Cortajarena A.L., Mejias S.H., Agut M., Nonell S., Flors C. (2013). Singlet Oxygen Generation by the Genetically Encoded Tag miniSOG. J. Am. Chem. Soc..

[24] Ruiz-González R., Bresolí-Obach R., Gulías O., Agut M., Savoie H., Boyle R.W., Nonell S., Giuntini F. (2017). NanoSOSG: A nanostructured fluorescent probe for the detection of intracellular singlet oxygen. Angew. Chem. Int. Ed..

[25] Bresolí-Obach R., Nos J., Mora M., Sagristà M.L., Ruiz-González R., Nonell S. (2016). Anthracene-based fluorescent nanoprobes for singlet oxygen detection in biological media. Methods.

[26] Ruiz-González R., Zanocco R., Gidi Y., Zanocco A.L., Nonell S., Lemp E. (2013). Naphthoxazole-Based Singlet Oxygen Fluorescent Probes. Photochem. Photobiol..

[27] Planas O., Bresolí-Obach R., Nos J., Gallavardin T., Ruiz-González R., Agut M., Nonell S. (2015). Synthesis, Photophysical Characterization, and Photoinduced Antibacterial Activity of Methylene Blue-loaded Amino- and Mannose-Targeted Mesoporous Silica Nanoparticles. Molecules.

[28] Schneider C.A., Rasband W.S., Eliceiri K.W. (2012). NIH image to imageJ: 25 years of image analysis. Nat. Methods.

[29] Schindelin J., Arganda-Carreras I., Frise E., Kaynig V., Longair M., Pietzsch T., Preibisch S., Rueden C., Saafeld S., Schmid B. (2012). Fiji: An open-source platform for biological-image analysis. Nat. Methods.

[30] Neises B., Steglich W. (1978). Simple Method for the Esterification of Carboxylic Acids. Angew. Chem. Int. Ed..

[31] Zanocco R.P., Bresoli-Obach R., Nonell S., Lemp E., Zanocco A.L. (2018). Structure-activity study of furyl aryloxazole fluorescent probes for the detection of singlet oxygen. PLoS ONE.

[32] Wilkinson F., Helman W.P., Ross A.B. (1995). Rate constants for the decay and reactions of the lowest electronically excited singlet state of molecular oxygen in solution. An expanded and revised compilation. J. Phys. Chem. Ref. Data.

[33] Clennan E.L., Mehrsheikh-Mohammadi M.E. (1984). Mechanism of endoperoxide formation. 3. Utilization of the Young and Carlsson kinetic techniques. J. Am. Chem. Soc..

[34] Denk W., Strickler J., Webb W. (1990). Two-photon laser scanning fluorescence microscopy. Science.

[35] Hoover E.E., Squier J.A. (2013). Advances in multiphoton microscopy technology. Nat. Photonics.

[36] Liu H.W., Xu S., Wang P., Hu X.X., Zhang J., Yuan L., Zhang X.B., Tan W. (2016). An efficient two-photon fluorescent probe for monitoring mitochondrial singlet oxygen in tissues during photodynamic therapy. Chem. Commun..

[37] Fletcher A.A. (1969). Quinine sulfate as a fluorescence quantum yield standard. Photochem. Photobiol..

[38] NIST Standard Reference Database 35. https://webbook.nist.gov/cgi/cbook.cgi?ID=C116165&Mask=80#IR-Spec.

[39] Günther G.S., Lemp E., Zanocco A.L. (2000). On the use of 9,10-dimethylanthracene as chemical rate constant actinometer in singlet molecular oxygen reactions. Bol. Soc. Chil. Quím..

[40] Schmidt R., Tanielian C., Dunsbach R., Wolff C., Womb C.J. (1994). Phenalenone, a universal reference compound for the determination of quantum yields of singlet oxygen O_2_(^1^Δ_g_) sensitization. Photochem. Photobiol. A Chem..

[41] Marti C., Jürgens O., Cuenca O., Casals M., Nonell S. (1996). Aromatic ketones as standards for singlet molecular oxygen photosensitization. Time-resolved photoacoustic and near-IR emission studies. J. Photochem. Photobiol. A Chem..

[42] Rappé A.K., Casewit C.J., Colwell K.S., Goddard W.A., Skid W.M. (1992). UFF, a Full periodic table force field for molecular mechanics and dynamics simulations. J. Am. Chem. Soc..

