# Supplementary material for: NanoFN10: A High-Contrast Turn-On Fluorescence Nanoprobe for Multiphoton Singlet Oxygen Imaging
Source: Sensors (Basel). 2023 May 9;23(10):4603. doi: 10.3390/s23104603 (PMC10222627; doi:10.3390/s23104603)
Supplement: Supplementary file 1 [file sensors-23-04603-s001.zip › sensors-2301260-supplementary.docx]

Electronic Supporting Information

NanoFN10: A high-contrast turn-on fluorescence nanoprobe for multiphoton singlet oxygen imaging

Renzo P. Zanocco^1,^*, Roger Bresolí-Obach,^2^ Francisco Nájera,^3,4^ Ezequiel Pérez-Inestrosa,^3,4^ Antonio L. Zanocco,^1^ Else Lemp,^1^ Santi Nonell^2,^*

Figure S1 Spectroscopic characterization of FN10.

Figure S2 Determination of the FN10 fluorescence quantum yield.

Figure S3 HPLC monitoring of FN10 oxidation by singlet oxygen.

Figure S4 Spectroscopic characterization of FN10-ox.

Figure S5 Evolution of the excitation spectra of FN10 upon reaction with singlet oxygen.

Figure S6 Reactivity of FN10 with singlet oxygen.

Figure S7 Reactivity of FN10 with other reactive oxygen species.

Figure S8 Quantum yield of singlet oxygen production by FN10.

Figure S9 Geometry of FN10 grafted onto the surface of a silica particle through a PEG linker.

Figure S10 Fluorescence evolution of the nanoFN10RB probe upon reaction with singlet oxygen.

Figure S11 Two-photon microscopy of N13 microglia cells incubated with nanoFN10RB.

Figure S12 Co-localization of FN10 and Rose Bengal fluorescence in microglia cells incubated with nanoFN10RB.

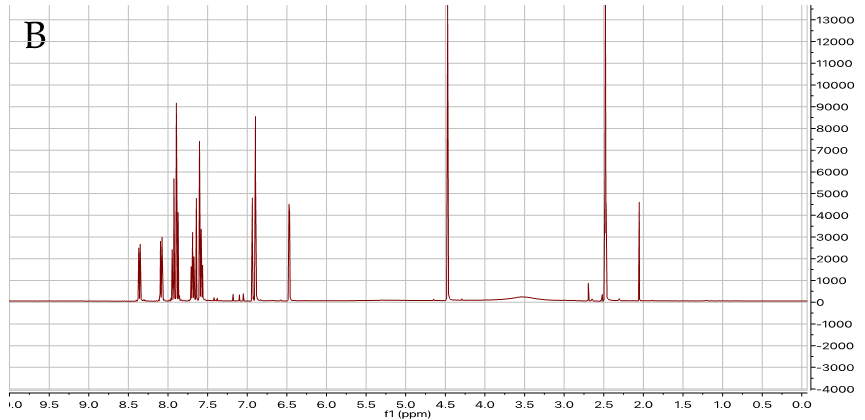


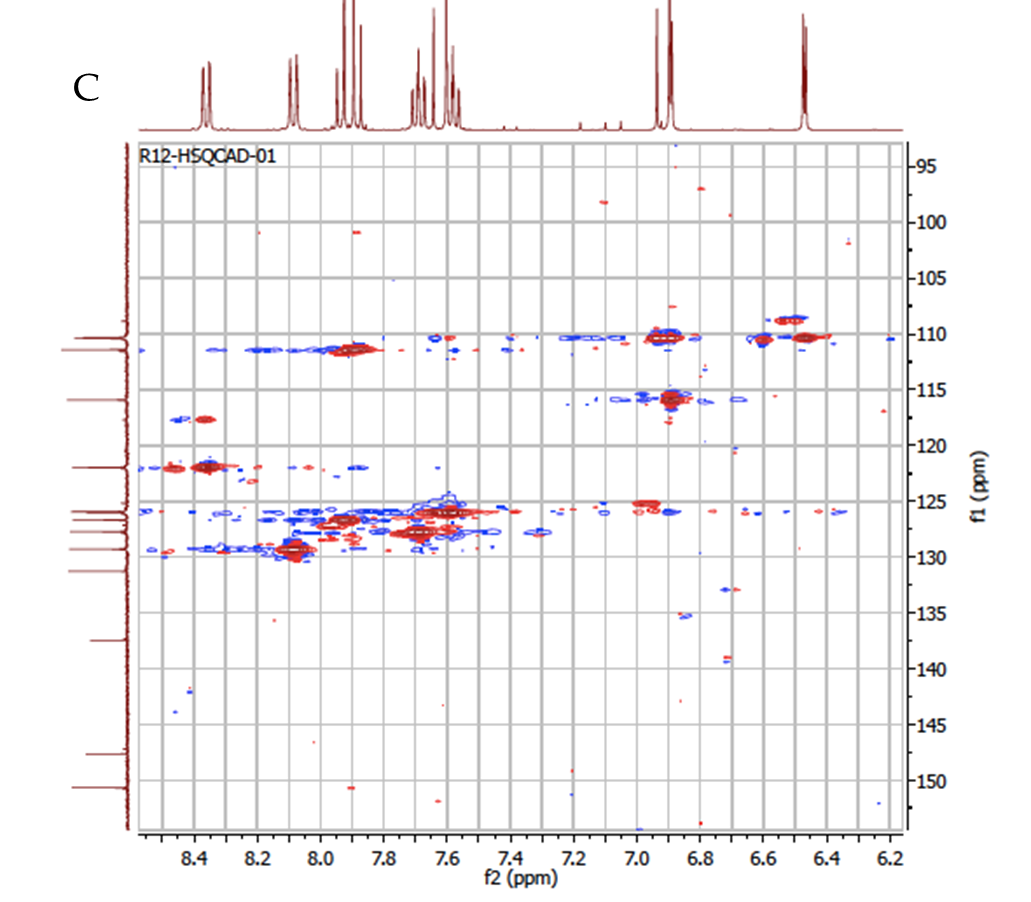


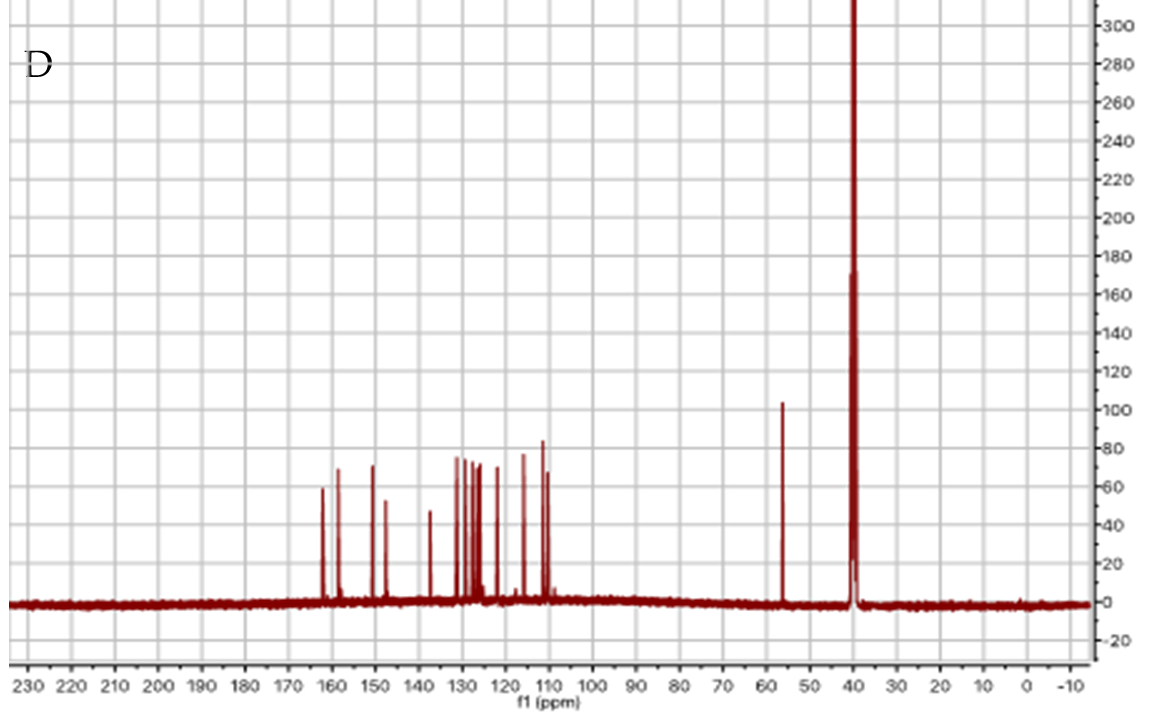

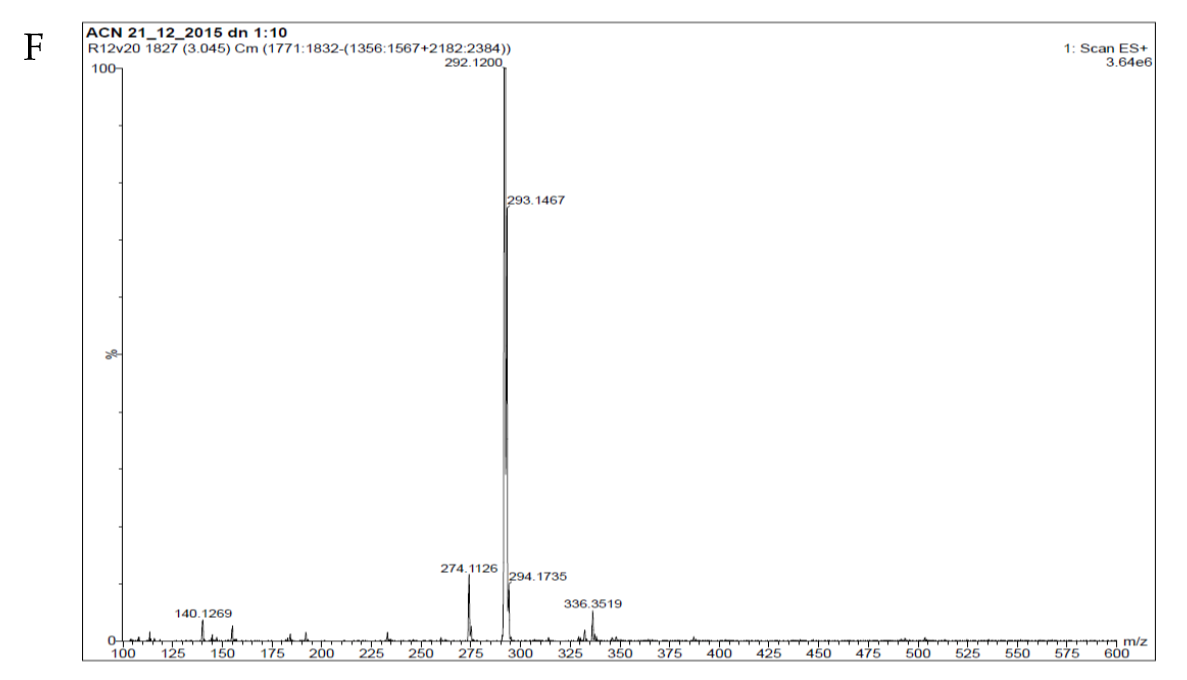


**Figure S1.** (A)Synthesis of the FN10. Spectroscopic characterization of FN10. (B) ^1^H-NMR spectrum in DMSO-d_6_. (C) ^13^C-NMR spectrum in DMSO-d_6_. (D) ^1^H-^13^C Heteronuclear single quantum coherence (HSQC) spectrum in DMSO-d_6_. (E) IR spectrum. (F) Mass spectra shows an intense signal with m/z = 292.12 Dalton.

**
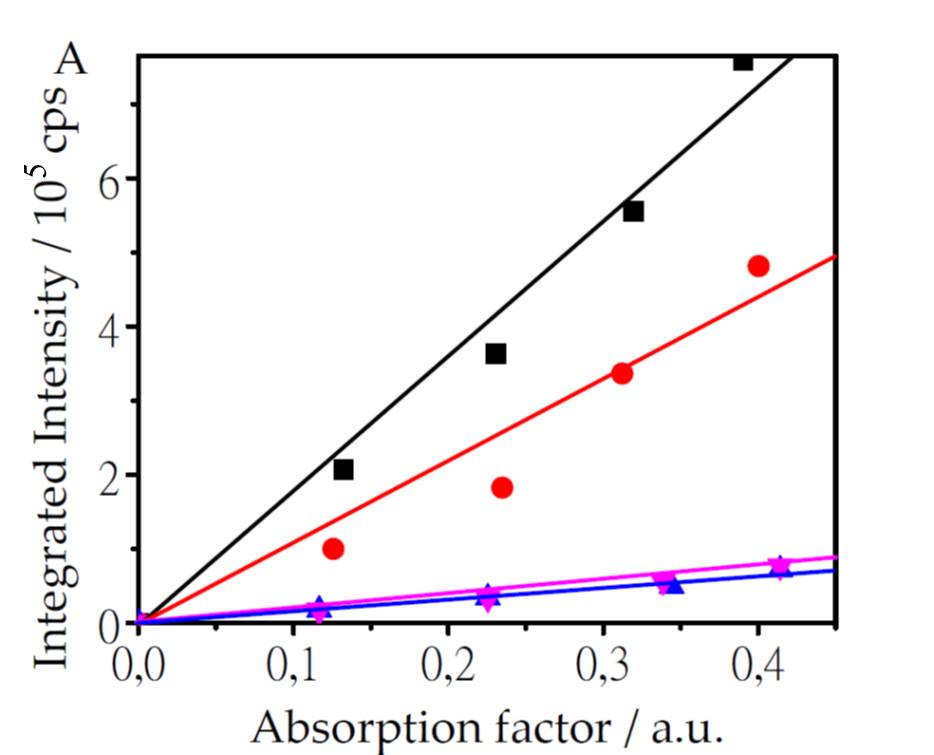
**

**Figure S2** Determination of the FN10 fluorescence quantum yield (Φ_F_). (A): Absorption-factor dependence of FN10 fluorescence intensity in different solvents: benzene (green dashed line), acetonitrile (red dashed line), *N,N’*-dimethylformamide (blue dashed line) and methanol (magenta dashed line). (B): Absorption-factor dependence of the fluorescence intensity of quinine sulphate in 0.1 M H_2_SO_4_, used as reference (Φ_F_ = 0.54)[38].

**Figure S3** HPLC monitoring of FN10 oxidation by singlet oxygen. (A): HPLC diode array detection chromatograms (C-18, 15 x 0.4 cm; CH_3_CN:CH_3_OH 90:10 as mobile phase); isocratic 0.8 mL/min) obtained for the reaction of FN10 with ^1^O_2_ as a function of photo-oxidation time between 0 (black line) and 40 min (purple line). (B): Changes in the relative intensity of the FN10 (red plot) and FN10-ox (blank plot) peaks. Photooxidation conditions: the photosensitizer was New Methylene Blue, the irradiation wavelength was 640 ± 10 nm and the experiment was carried out in aerated methanol.

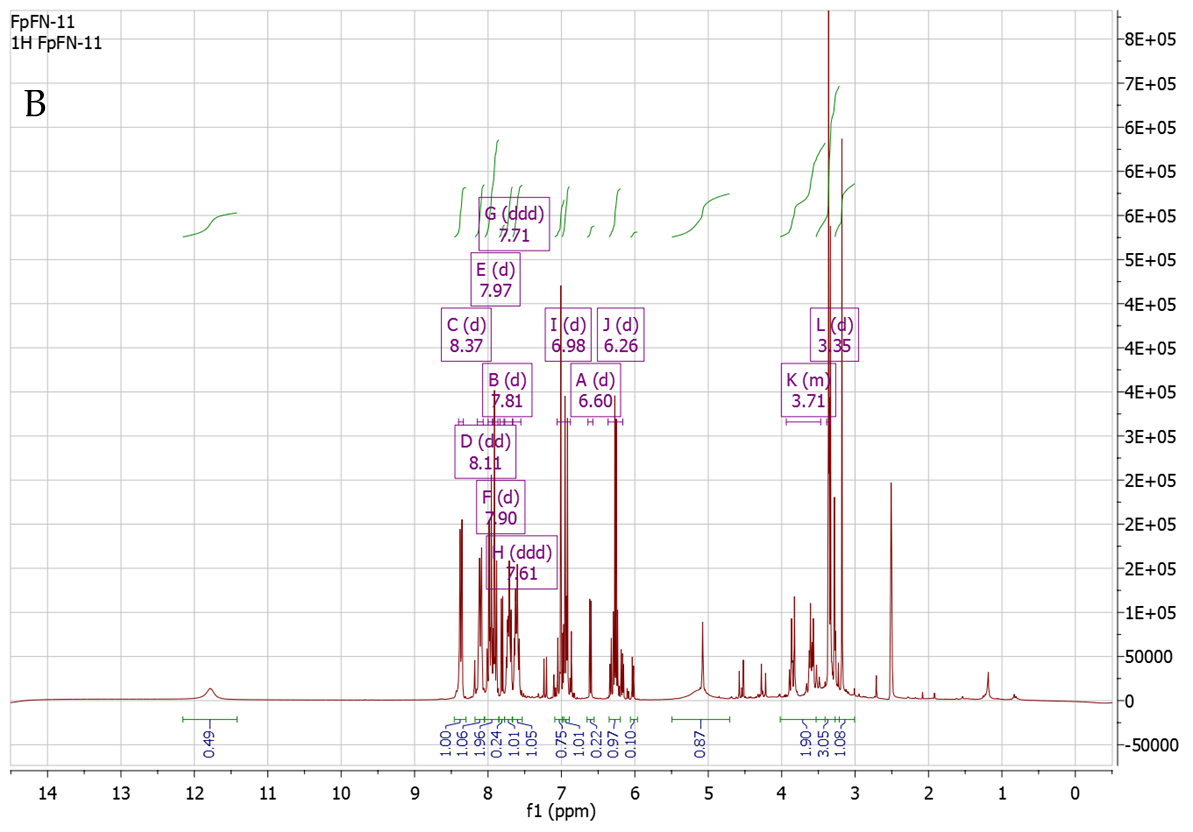


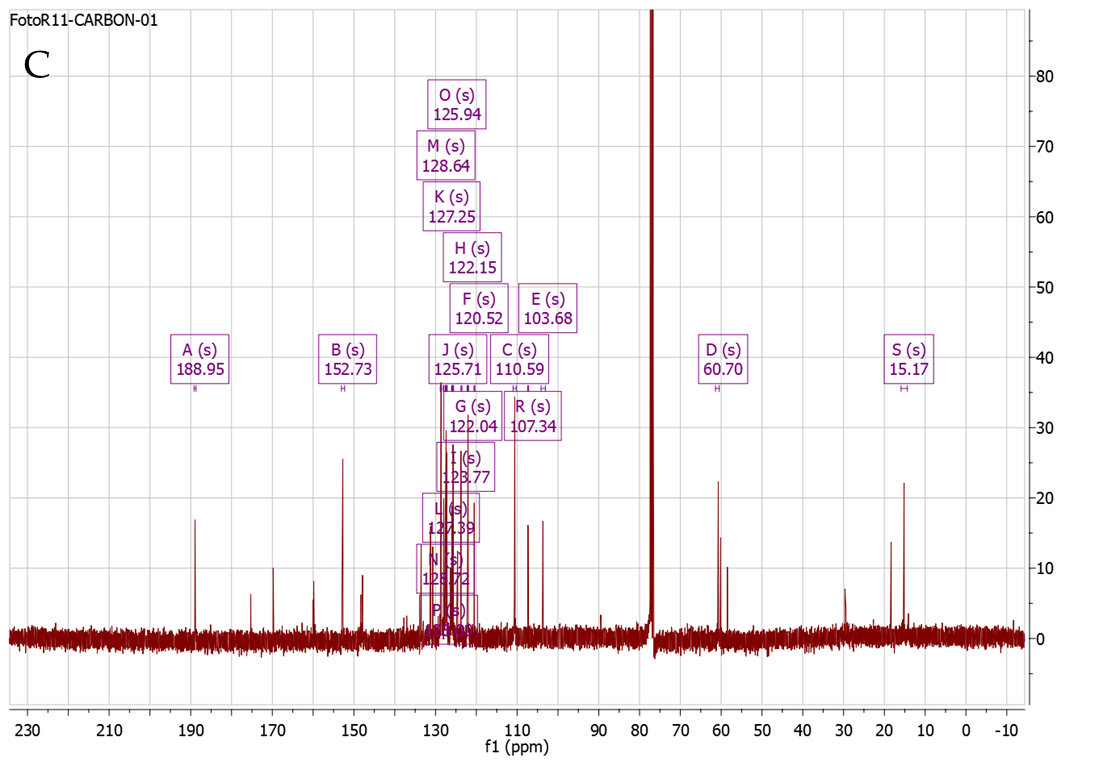

**Figure S4** Spectroscopic characterization of FN10-ox. (A) The IR spectrum obtained in film shows important signals at 3334, 3067, 2925, 1773, 1622, 1445 and 1083 cm^-1^. The most noteworthy signal, at 1773 cm^-1^, can be ascribed to the carbonyl groups of the photoproduct. The attractor effect, resulting from the π deficient nature of aryloxazole, can generate a center of positive charge on the carbon next to one of the ketone groups. In addition, the inductive attractor effect of the terminal methoxy group, it is likely to produce an electron deficient site in the vinyl carbon. The sum of these effects causes the conjugated carbonyl system to be electronically unprotected, explained why the IR signal of the carbonyl groups appears at higher energy than expected, such as occurs in hexachloroacetone whose IR signal is at 1770 cm^‑1^ [39]. (B): ^1^H-NMR spectrum in DMSO-d_6_. (C): ^13^C-NMR spectrum in CD_3_Cl. Both spectra show a set of signals corresponding to solvent residues and minor decomposition products, which could not be separated, as well as signals corresponding to the dicarbonyl and enol tautomers (simulation superimposed as magenta lines. (D): Mass spectra shows an intense signal with m/z = 322.36 Dalton.

**Figure S5** Evolution of the excitation spectra of FN10 upon reaction with singlet oxygen (λ_obs_ = 450 nm). The photosensitizer was New Methylene Blue, the irradiation wavelength was 640 ± 10 nm and the experiment was carried out in aerated methanol.

|  |
| --- |
| **** |

**Figure S6** Reactivity of FN10 with singlet oxygen. (A) Singlet oxygen quenching (lifetime decrease) in the presence of increasing FN10 concentrations; excitation wavelength 620 nm, solvent MeOH, sensitizer NMB. (B): Determination of the rate constant for singlet oxygen quenching by FN10 (*k*_q_) from measurements of the singlet oxygen lifetime in solutions with increasing concentrations of FN10. (C): Determination of the rate constant for the reaction of singlet oxygen with FN10 (*k*_r_; dashed black line) by comparing the rates of absorbance loss for FN10 (observation wavelength; 360 nm) and the reference substrate 9,10-dimethylanthracene (observation wavelength; 380 nm, solid grey line; *k*_r_ = (6.3 ± 0.4) x 10^7^ M^-1^s ^-1^) according to the procedure outlined in reference [40]. Solvent: methanol, photosensitizer: New Methylene Blue.

**Figure S7** Reactivity of FN10 with other reactive oxygen species**.** Effect of 10-fold excess of (A) superoxide radical anion and (B) hydrogen peroxide on the absorbance of an FN10 solution in methanol.

**Figure S8** Quantum yield of singlet oxygen production by FN10. The quantum yield of singlet oxygen production (Φ_Δ_) was determined in (A) acetonitrile and (B) methanol by comparing the intensity of singlet oxygen emission at 1275 nm [32] for FN10 (red lines) and the reference photosensizer phenalenone (black lines; (Φ_Δ_ ~ 1.0 in both solvents) [41,42] upon pulsed-laser excitation at 355 nm.


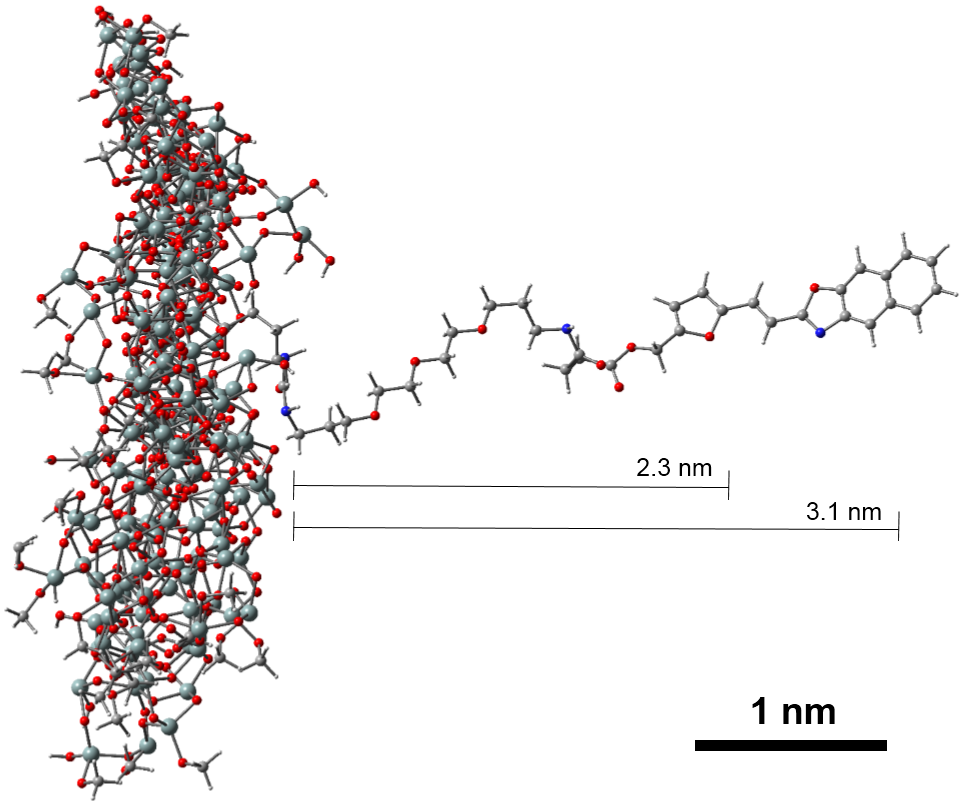


**Figure S9** Geometry of FN10 grafted onto the surface of a silica particle through a PEG linker. Modelling was carried out using Molecular Mechanics with the Universal Force Field (UFF). [43]

**Figure S10** Fluorescence evolution of the nanoFN10RB probe upon reaction with singlet oxygen. (A): nanoFN10RB emission spectra changes upon reaction with ^1^O_2_ (λ_exc_ = 330 m). The irradiation wavelength was 560 ± 10 nm and the experiment was carried out in aerated methanol. (B): nanoFN10RB fluorescence enhancement evolution at λ_obs_ =390 nm.

**Figure S11** Two-photon microscopy of N13 microglia cells incubated with nanoFN10RB. (A): X-Y fluorescence image (λ_exc_ = 720 nm) in standard culture medium. The white cross indicates the location of the axial planes studied. (B): Fluorescence image in the Y-Z perpendicular plane. (C): Fluorescence image in the X-Z perpendicular plane. Scale bar: 10 μm.

**Figure S12** Co-localization of FN10 and Rose Bengal fluorescence in N13 microglia cells incubated with nanoFN10RB. (A): Two-photon microscopy image of FN10 (λ_exc_ = 720 nm); (B): One-photon microscopy image of RB (λ_exc_ = 561 nm; λ_obs_ = 580-700 nm); (C): overlapping images from (A) and (B). Scale bar: 20 μm.
